# ciliaFA: a research tool for automated, high-throughput measurement of ciliary beat frequency using freely available software

**DOI:** 10.1186/2046-2530-1-14

**Published:** 2012-08-01

**Authors:** Claire M Smith, Jana Djakow, Robert C Free, Petr Djakow, Rana Lonnen, Gwyneth Williams, Petr Pohunek, Robert A Hirst, Andrew J Easton, Peter W Andrew, Christopher O’Callaghan

**Affiliations:** 1 Department of Infection, Immunity and Inflammation, University of Leicester, University Road, Leicester LE1 9HN, UK; 2 Department of Paediatrics, Second Faculty of Medicine, University Hospital Motol, Prague, Czech Republic; 3 Department of Genetics, University of Leicester, University Road, Leicester LE1 9HN, UK; 4 Siemens IT Solutions, Prague, Czech Republic; 5 Department of Biological Sciences, University of Warwick, Warwick CV4 7AL, UK

## Abstract

**Background:**

Analysis of ciliary function for assessment of patients suspected of primary ciliary dyskinesia (PCD) and for research studies of respiratory and ependymal cilia requires assessment of both ciliary beat pattern and beat frequency. While direct measurement of beat frequency from high-speed video recordings is the most accurate and reproducible technique it is extremely time consuming. The aim of this study was to develop a freely available automated method of ciliary beat frequency analysis from digital video (AVI) files that runs on open-source software (ImageJ) coupled to Microsoft Excel, and to validate this by comparison to the direct measuring high-speed video recordings of respiratory and ependymal cilia. These models allowed comparison to cilia beating between 3 and 52 Hz.

**Methods:**

Digital video files of motile ciliated ependymal (frequency range 34 to 52 Hz) and respiratory epithelial cells (frequency 3 to 18 Hz) were captured using a high-speed digital video recorder. To cover the range above between 18 and 37 Hz the frequency of ependymal cilia were slowed by the addition of the pneumococcal toxin pneumolysin. Measurements made directly by timing a given number of individual ciliary beat cycles were compared with those obtained using the automated ciliaFA system.

**Results:**

The overall mean difference (± SD) between the ciliaFA and direct measurement high-speed digital imaging methods was −0.05 ± 1.25 Hz, the correlation coefficient was shown to be 0.991 and the Bland-Altman limits of agreement were from −1.99 to 1.49 Hz for respiratory and from −2.55 to 3.25 Hz for ependymal cilia.

**Conclusions:**

A plugin for ImageJ was developed that extracts pixel intensities and performs fast Fourier transformation (FFT) using Microsoft Excel. The ciliaFA software allowed automated, high throughput measurement of respiratory and ependymal ciliary beat frequency (range 3 to 52 Hz) and avoids operator error due to selection bias. We have included free access to the ciliaFA plugin and installation instructions in Additional file 1 accompanying this manuscript that other researchers may use.

## Background

In the human respiratory tract cilia beat in a coordinated fashion at a frequency of approximately 10 to 14 Hz, propelling mucus towards the pharynx where it is swallowed [1]. This process is known as mucociliary clearance (MCC). In the brain, ependymal cilia beat at a frequency of around 40 Hz, moving cerebrospinal fluid (CSF) close to the ventricular wall. Many factors, including inherited ciliary defects in primary ciliary dyskinesia (PCD), temperature, pH, viscosity and exposure to bacterial and viral pathogens have been shown affect ciliary function [2-5]. Recent studies have shown that assessment of both ciliary beat pattern and frequency are essential as cilia may beat in a dyskinetic fashion while maintaining their normal beat frequency [5-7]. The most accurate method for determining ciliary beat frequency, particularly at high beat frequencies is by directly timing a given number of individual ciliary beat cycles from slow-motion playback of high-speed video files [7]. However, this is extremely time consuming and automated methods designed to rapidly measure beat frequency from high-speed video recordings are needed [8-10].

**Figure 1:**
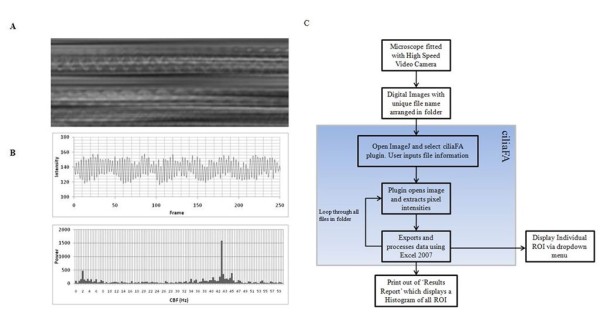
**(A) Diagrammatic view of the change in light intensity surrounding motile cilia constructed using the Volume Viewer plugin for ImageJ. (B)** Rhythmic changes in light intensity are extracted as pixel intensity over time/frame, which is the raw data used to obtain cilia beat frequency. **(C)** Flow chart of the ciliaFA and conventional methods used to calculate ciliary beat frequency (CBF) from digital video files.

Early automated methods, including the laser light-scattering spectroscopy, photodiode and photomultiplier methods were developed to calculate ciliary beat frequency (CBF) based on changes in light intensity around beating cilia under the microscope [11-13]. More recently, a number of bespoke image analysis software programs have been developed to automatically compute CBF from video files captured using video microscopy [8-10]. This software computes changes in pixel intensity over time (as shown in Figure 1A) to Hertz using fast Fourier transformation (FFT), a function that has been used frequently for this application [14,15]. However, the studies that describe this software lack two important elements: comparison of their results to the direct measurement of ciliary beat frequency from slow-motion video replay of cilia, and the measurement cilia that beat at very high frequencies [8,9]. This is important as some phenotypes of PCD display respiratory cilia that beat at frequencies above 16 Hz and brain ependymal cilia beat at frequencies higher than 36 Hz. In this paper, we describe software that was validated by comparing results to those obtained by direct measurement of high-speed video recordings of cilia. The program also runs using open-source software. To evaluate a wide range of ciliary beat frequencies we studied both human respiratory cilia and ependymal cilia from the rat brain. Respiratory cilia usually beat at less than 16 Hz [7] and brain cilia usually beat at over 36 Hz. To obtain CBF measurements above 16 Hz, we slowed the frequency at which ependymal cilia beat by adding the pneumococcal toxin pneumolysin, which we have previously shown to reduce ependymal CBF [4]. The ciliaFA program has been made freely available and may be downloaded in Additional file 1.

## Methods

### Ependyma tissue preparation

Vibratome sections (250 μm thick) of the floor of the fourth ventricle of the brains of infant Wistar rats (between 9 and 15 days of age) were prepared to allow ependymal cilia to be viewed on an inverted microscope using a × 40 objective lens. Each section was submerged under 4 ml of media 199 (M199, Gibco, Invitrogen, Paisley, UK), as described previously [16].

### Human respiratory cilia

Human respiratory epithelium was obtained by brushing the inferior nasal turbinate with a 2-mm cytology brush (Keymed, Southend-on-Sea, UK) as previously described [7]. Cells were dislodged from the brush into M199 medium and grown to a ciliated phenotype at air-liquid interface as previously described [17]. Ethical approval for the collection of nasal epithelial cells was given by the Leicestershire Ethical Review Committee.

### Measurement of CBF

To determine ciliary beat frequency, both the brain slices and the respiratory cells in culture were placed in an incubation (37°C) chamber and were observed via an inverted microscope system (Nikon TU1000, Kingston-upon-Thames, UK). Tissue was allowed to equilibrate for 30 minutes before readings. Beating cilia were recorded using a Motion Pro X4 digital high-speed video camera (Lake Image Systems, Henrietta, N.Y. USA) at a rate of 250 to 500 frames per second using an × 40 objective as previously described [18]. At least 512 or 1,024 frames were captured, respectively.

The camera allows video sequences to be recorded and played back at reduced frame rates or frame by frame. CBF is calculated by the observer timing a given number of individual cilia beat cycles using the following equation: Frame rate (number of frames/sec)/5 (frames elapsed for five ciliary beat cycles) × 5 (conversion per beat cycle).

### Inhibition of CBF

To evaluate beat frequencies between the upper limit of normal (approximately 16 Hz) for respiratory cilia and the lower limit of normal for ependymal cilia (approximately 36 Hz) we slowed ependymal ciliary beat frequency by the addition of the pneumococcal toxin, pneumolysin. We have previously shown pneumolysin to reduce ependymal ciliary beat frequency [4]. Pneumolysin was purified as previously described [19]. Cells were exposed to 1 ml M199 containing 300 ng of pneumolysin, which was preheated to 37°C. The CBF was measured at intervals of 0, 5, and 15 minutes over the course of the experiment. At each time point, images were captured from four different areas along the ciliated edge of the brain slice.

### Software development

To develop software to batch-process and calculate CBF, we focused on two core components: the first was to extract the pixel intensities of particular region of interest (ROI) over time and the second to use this data for fast Fourier transformation (FFT). We used the freely available open-source ImageJ software to determine the average pixel intensity of up to 40 × 40 ROI per frame of the AVI file [20]. The ciliaFA plugin exports a dataset of up to 1,600 ROI to Excel 2007 (Microsoft; Redmond, WA, USA) where a visual basic macro is initiated to perform the FFT. The complex number is translated using the Excel function ‘=IMABS(range)’ and the dominant frequency within the range is then established using the function ‘=MAX(range)’. The CBF is determined by multiplying the row number by the frequency resolution (FR) (frame rate of recording/number of frames), which we named the ‘FFT Mag’. In order to more accurately predict the CBF, we averaged the sum of the FFT Mag of the peaks flanking the maximum (see Figure 1B).

### Noise reduction

We found that excluding data based on the following criteria significantly reduced the effect of background interference that can result from Fourier analysis: (1) the amplitude of the peak must be greater than three times the amplitude of the background (defined as the maximum peak of the first three FFT Mag), (2) the CBF must be within a clinically relevant range (for example between 3 to 20 Hz for respiratory cilia and 3 to 60 Hz for brain ependymal cilia). Data that does not support these criteria was defined as 0 Hz.

**Figure 2:**
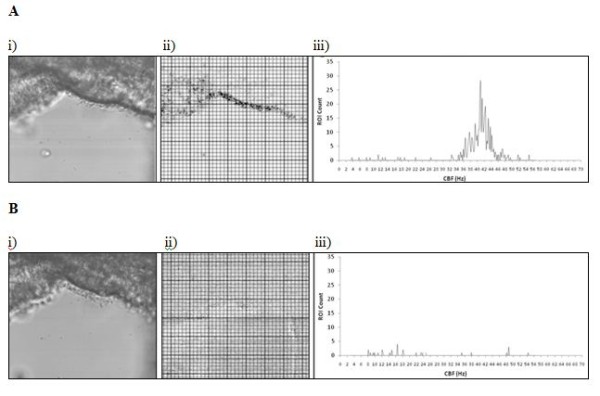
**Data analysis of one.** AVI file of ependymal cilia before (**A**), and 15 minutes (**B**) after the addition of the bacterial toxin, pneumolysin. Images show (i) the area of brain tissue investigated, (ii) the regions of interest (ROI) used for analysis (color coding shows the intensity of the ciliary beat frequency (CBF) for each ROI: the darker the displayed color, the higher the CBF) and (iii) a histogram of the CBF of all regions of interest.

The resulting Excel data sheet presents the CBF calculations as a report that allows the observer to obtain information about individual regions of interest or the image as a whole. Raw data (including pixel intensities and the power spectrum of the FFT (Figure 1B)) from individual regions of interest can be inspected using a drop-down menu. The results report displays the mean, median and modal CBFs of the field, and a histogram of the CBF of all regions of interest (Figure 2A). A color chart of the intensity of the CBF for each regions of interest is also presented in Figure 2B.

### Statistical analysis

Linear regression was used to correlate CBF measurements made by conventional frame by frame counting of individual ciliary beat cycles by slow-motion playback of digital high-speed video sequences to measurements obtained using the ciliaFA system (for the same region of interest). All regions of interest were chosen based on areas where optimal images of moving cilia were visible to the observer. The video sequences were reanalyzed by a second observer. Paired t tests were performed to compare the CBF obtained using the two methods and the Bland-Altman limits of agreement were calculated from the mean difference ± the 95% confidence intervals between the two methods.

## Results

**Figure 3:**
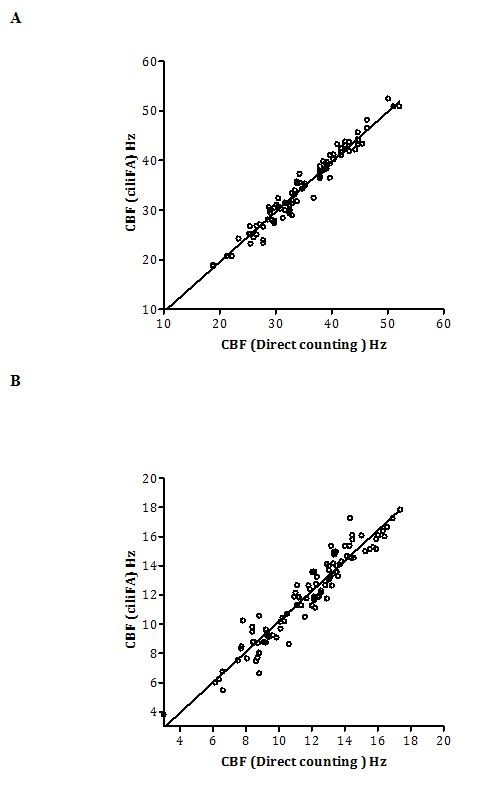
Linear regression of the mean ciliary beat frequency (CBF) measurements by conventional frame by frame counting of individual ciliary beat cycles (direct counting method) compared with the ciliaFA system (A) using ependymal cilia (n = 100) and (B) respiratory cilia (n = 100) at 37°C.

A total of 200 measurements were made for ciliary beat frequency, 100 for ependymal cilia and 100 for respiratory cilia. There was no significant difference (paired *t* test, *P* = 0.64) in the ciliary beat frequency obtained using the direct counting and ciliaFA methods (n = 200). The mean difference (± SD) was −0.05 ± 1.25 Hz and was highly correlated (r^2^ = 0.991) (Figure 3).

For ependymal cilia the mean CBF was 36.3 ± 6.4 Hz for the direct counting and 35.9 ± 7.0 Hz for the ciliaFA method. For respiratory cilia the mean CBF was 11.72 ± 2.8 Hz for the direct counting and 11.97 ± 3.0 Hz for the ciliaFA method. The Bland-Altman limits showed agreement from −2.55 to 3.25 Hz for the ependymal cilia and showed a closer agreement for the respiratory cilia (−1.99 to 1.49 Hz).

The addition of the bacterial toxin pneumolysin reduced the mean CBF of ependymal cilia within 5 minutes (Table 1). The mean CBF values after 5 minutes exposure to pneumolysin determined by the ciliaFA was 21.45 ± 4.1 Hz. Using the direct counting method, two different observers calculated a mean CBF of 21.75 ± 4.5 Hz and 21.79 ± 4.2 Hz respectively. Repeatability (agreement between methods) was very good and presented values similar to the agreement between two different observers (1.13 Hz and 1.08 Hz respectively). At 15 minutes after the addition of pneumolysin the cilia were mostly static (Figure 2). The ciliaFA accurately predicted the reduction in CBF and detected regions of interest where the cilia had become static.

**Table 1:** The ependymal ciliary beat frequency (CBF) determined by two observers using high-speed digital imaging and the ciliaFA software

**Time**	**CBF (Hz)**			
	**ciliaFA**	**Direct counting, observer 1**	**Direct counting, observer 2**	**Observer 1 (second count)**
0 minutes	43.05	39.06	42.37	47.17
	42.55	37.87	41.67	40.32
	45.25	43.85	44.64	44.64
	42.40	39.68	41.67	39.68
	43.07	41.66	42.37	41.67
	43.31	38.46	40.98	46.29
	39.80	33.78	37.88	39.68
	40.30	30.86	32.00	30.86
5 minutes	18.86	18.79	18.79	18.25
	24.28	23.36	23.15	23.15
	20.78	21.32	22.52	24.75
	18.97	18.79	18.66	18.66
	18.71	18.79	18.51	18.94
	18.82	18.79	18.66	18.51
	20.77	22.12	23.15	22.52
	30.43	32.00	30.86	30.86
ciliaFA-observer variability		1.42 Hz	0.84 Hz	-
Interobserver variability			1.17 Hz	-
Intraobserver variability				1.08 Hz

The effect of the addition of the bacterial toxin pneumolysin was monitored over time. Data are from eight regions of interest (ROI) at each time point.

## Discussion

We have developed a plugin for ImageJ that calculates CBF from digital AVI files recorded using high-speed video microscopy. As opposed to previous studies we validated the software by comparing the results to frequencies obtained using the most accurate method for CBF calculation, directly counting individual ciliary beat cycles during slow-motion playback. Our data showed that the software was validated for use over a wide range of ciliary beat frequencies up to 52 Hz. Table 1 shows that the limits of agreement between the two methods were within the same range as the natural variation that exists between two different observers. Furthermore, the Bland-Altman limits of agreement for human respiratory cilia between the ciliaFA and direct counting methods were very low. Previous software programs validated their results using indirect, less accurate methods, including the early automated photometry method [9]. Previous studies reported limits of agreement ranging from −1.0 to 1.39 Hz [9], -2.75 to 5.15 Hz [7] and from −3.89 to 3.39 Hz [8] compared to digital high-speed methods. In our system the image capture instrument is not connected to the software, so analysis performed once the experiment is completed. This allows the software to batch process groups of images using the same settings and thus reducing operator input.

This study has clearly demonstrated that ciliaFA is reliable system to analyze CBF. In addition to the accurate measurement of ciliary beat frequency the ciliaFA system has a number of advantages. It has high throughput capabilities allowing significant time savings when processing large datasets, it reduces operator error due to selection bias, and it accurately reports static cilia. The latter is a significant advantage as immotile cilia may not be visible to the observer in multilayered cell cultures. Furthermore, by focusing on particular ciliated areas, ciliaFA can report changes and reductions in ciliary beat frequency.

Limitations of the software depend largely on image quality (pixel size) and the number of frames captured. We have used the ciliaFA software to examine AVI files of different lengths, captured at different frame rates (data not shown). We found that videos captured using high-speed video cameras that capture at rates at least 120 frames per second with a length of 128 frames (to give a frequency resolution of 0.94) will give valid data. These settings capture an appropriate number of ciliary beat cycles to accurately average the CBF; the lower the frequency resolution (that is, the more frames captured), the greater the accuracy of CBF. To allow the videos to be used for beat pattern analysis high-speed video cameras that capture at rates exceeding 250 frames per second should be used. The ciliaFA software has the capability to crop frames from large files to speed up the calculation of frequency without reducing the frequency resolution. For high-frequency analysis, we recommend that the microscope bulb is connected to a stable power source of at least 110 volt AC/60 Hz; lower voltages may cause the bulb to flicker within the CBF range and this will enhance background noise. We also recommend that the image is not subject to downstream processing, such as enhanced pixel gain, as this will also enhance background noise.

The open-source software (ImageJ) and Microsoft Excel platforms were chosen due to their ubiquity, low cost and ease of manipulation. We have included the ciliaFA plugin and installation instructions in Additional file 1 accompanying this manuscript, so it is free to use by other research groups.

## Conclusions

We have developed a freely available, automated method of CBF analysis from digital video (AVI) files that runs using open-source software (ImageJ) coupled to Microsoft Excel. The ciliaFA software allowed automated, high throughput measurement of respiratory and ependymal ciliary beat frequency over a wide range of frequencies (range 3 to 52 Hz) and avoids operator error due to selection bias.

## Competing interests

The authors declare they have no competing interests.

## Authors’ contributions

CMS conceived of the study and cocreated the software, acquired and analyzed the data, and assembled the manuscript. JD conceived of the study and cocreated the software, analyzed the data, and contributed to the assembly of the manuscript. RCF contributed to software design. PD contributed to software design. RL provided purified pneumolysin. GW prepared primary human respiratory cell cultures. PP contributed to review and final approval of the manuscript. RAH contributed to review and final approval of the manuscript. AJE contributed to review and final approval of the manuscript. PWA contributed to analysis of the data and final approval of the manuscript. CO’C contributed to the conception of the study, analysis of the data, and review and final approval of the manuscript. All authors read and approved the manuscript.

## Supplementary Material

Additional file 1 **This folder contains all the files needed for successful installation of the ImageJ plugin ‘ciliaFA’.** These include: the ciliaFA installation guide, a ciliaFA free software license agreement, the ciliaFA.txt and ExcelRunTest.java program files, and the Excel files needed to process the data. Click here for file
