# Supplementary material for: ciliaFA: a research tool for automated, high-throughput measurement of ciliary beat frequency using freely available software
Source: Cilia. 2012 Aug 1;1:14. doi: 10.1186/2046-2530-1-14 (PMC3607980; doi:10.1186/2046-2530-1-14)
Supplement: Additional file 1 — This folder contains all the files needed for successful installation of the ImageJ plugin ‘ciliaFA’. These include: the ciliaFA installation guide, a ciliaFA free software license agreement, the ciliaFA.txt and ExcelRunTest.java program files, and the Excel files needed to process the data. [file 2046-2530-1-14-S1.zip › ciliaFA/CiliaFA FREE SOFTWARE LICENSE AGREEMENT.docx]

**CiliaFA FREE SOFTWARE LICENSE AGREEMENT**

By downloading the software you indicate your agreement with this disclaimer.

Permission to use, copy, modify, and distribute this software for any purpose without fee is hereby granted, provided that the manuscript is referenced and this permission notice appears intact in all copies of the software and that you do not sell the software, or include the software in a commercial package.

THE SOFTWARE IS PROVIDED "AS IS" AND THE AUTHOR DISCLAIMS ALL WARRANTIES WITH REGARD TO THIS SOFTWARE INCLUDING ALL IMPLIED WARRANTIES OF MERCHANTABILITY OR FITNESS FOR A PARTICULAR PURPOSE. The author also disclaims any future responsiblity for software support or maintenance.

IN NO EVENT SHALL THE AUTHOR BE LIABLE FOR ANY SPECIAL, DIRECT, INDIRECT, OR CONSEQUENTIAL DAMAGES OR ANY DAMAGES WHATSOEVER RESULTING FROM LOSS OF USE, DATA OR PROFITS, WHETHER IN AN ACTION OF CONTRACT, NEGLIGENCE OR OTHER TORTIOUS ACTION, ARISING OUT OF OR IN CONNECTION WITH THE USE OR PERFORMANCE OF THIS SOFTWARE.

The software is experimental ONLY and has not been designed for, tested or approved for clinical use. The use of the software is at the user's own risk. Any conclusions you may draw based on the software or its use are your own.
