# Supplementary material for: ciliaFA: a research tool for automated, high-throughput measurement of ciliary beat frequency using freely available software
Source: Cilia. 2012 Aug 1;1:14. doi: 10.1186/2046-2530-1-14 (PMC3607980; doi:10.1186/2046-2530-1-14)
Supplement: Additional file 1 — This folder contains all the files needed for successful installation of the ImageJ plugin ‘ciliaFA’. These include: the ciliaFA installation guide, a ciliaFA free software license agreement, the ciliaFA.txt and ExcelRunTest.java program files, and the Excel files needed to process the data. [file 2046-2530-1-14-S1.zip › ciliaFA/Installation Guide for ciliaFA ImageJ plugin.pdf]

Installation Guide

for the ImageJ plugin - *ciliaFA*

By Dr Claire M. Smith

If this software is used in future publications please reference us in your article:

CM. Smith, J Djakow, RC. Free, P Djakow, R Lonnen, G Williams, P Pohunek, RA. Hirst, AJ. Easton, PW. Andrew, CO'Callaghan. ciliaFA: A research tool for automated, high-throughput measurement of ciliary beat frequency using freely available software. Cilia, (2012)

If you want to thank us, please donate to our sponsors:

http://www.action.org.uk/support_us/donate_online

1. How to configure Excel to run ciliaFA

****Administrator rights are required for installation****
****Excel 2007 required****

You should use Excel 2007 or later
Setup Excel as follows:

1.1 Display developer ribbon
- Go to Excel Office Button
– Select “**Excel Options**”
– Check “**Show developer tab in Ribbon**”

1.2 Install the Analysis Toolpak and Analysis Toolpak VBA Addin.
- Go to Excel Office Button
- Select “**Excel Options**”
- go to “**Addins**” tab
- Manage “**Excel Addins**”. Click Go
- Select “**Analysis Toolpak**” and “**Analysis Toolpak VBA**” (shown below)
- Click OK

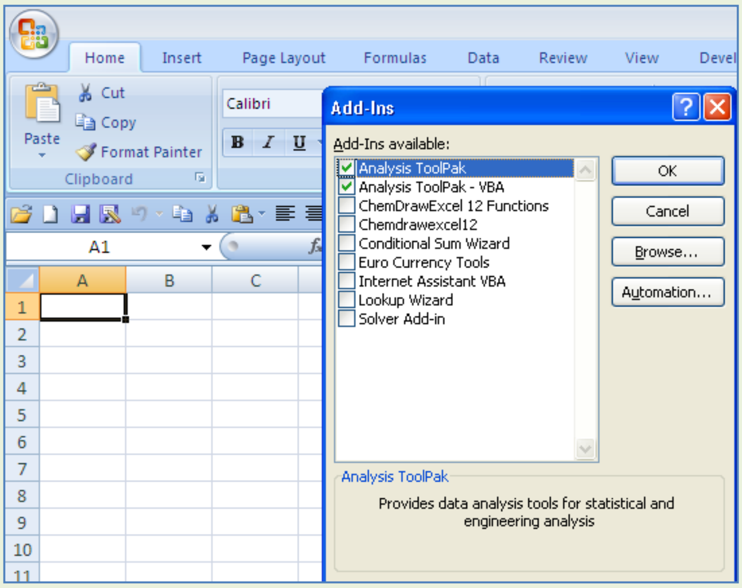

1.3 Disable Macro security
-Select “**Developer tab**”
-Then “**Macro security**”
-Then “**Macro Settings**”
- Check “**Enable all Macros**”

1.4 Close all programs

OPTIONAL: Minimising the Excel ribbon also increases the processing speed. Right click on the ribbon to change this setting.

2. How to install ciliaFA using ImageJ

2.1 Download and install ImageJ from http://rsbweb.nih.gov/ij/download.html

2.2 Choose ImageJ bundled with Java

2.3 Once ImageJ is installed, copy the whole unzipped "**ciliaFA**" folder into the plugins folder of ImageJ

i.e go to C:\Program Files\ImageJ\plugins

Then:

2.4 Open ImageJ

2.5 Go to "**Plugins**" tab, Select "**Compile and Run**"

2.6 Browse for the ciliaFA files in the plugins folder on the computer and choose the file "**ExcelRunTest.java**".

NB: If the file does not have the .java file extension showing, check the following.

Go to Folder Options and make sure the option to 'show file extensions' is checked.

2.7 Wait for the **"ExcelRunTest.class"** file to appear in your ciliaFA folder

2.8 Restart your computer

OPTIONAL: Change the ImageJ ROI grid colour to black

-Select "**Analyse**", then "**Tools**", then "**ROI Manager…**"
-Click on "**More**", and then "**Options**"
-Change "**Show All Color**" to Black by sliding all the bars to the left.

Now you are ready to use ciliaFA. If set up correctly, you should not need to re-install the software.

3. How to use ciliaFA

3.1 Group all your .avi files to be analysed in one folder. Make sure this folder only contains .avi files. Rename each .avi file so that it has a logical name that you can easily trace and understand. Each file name must be unique. All .avi files must start with a letter not a number, must be no longer than 16 characters and contain no punctuation.

3.2 When you are ready to begin analysis, select “**ciliaFA**” from the “**Plugins**” tab of ImageJ

3.3 This window then opens to allow you to input all the information required about your files:

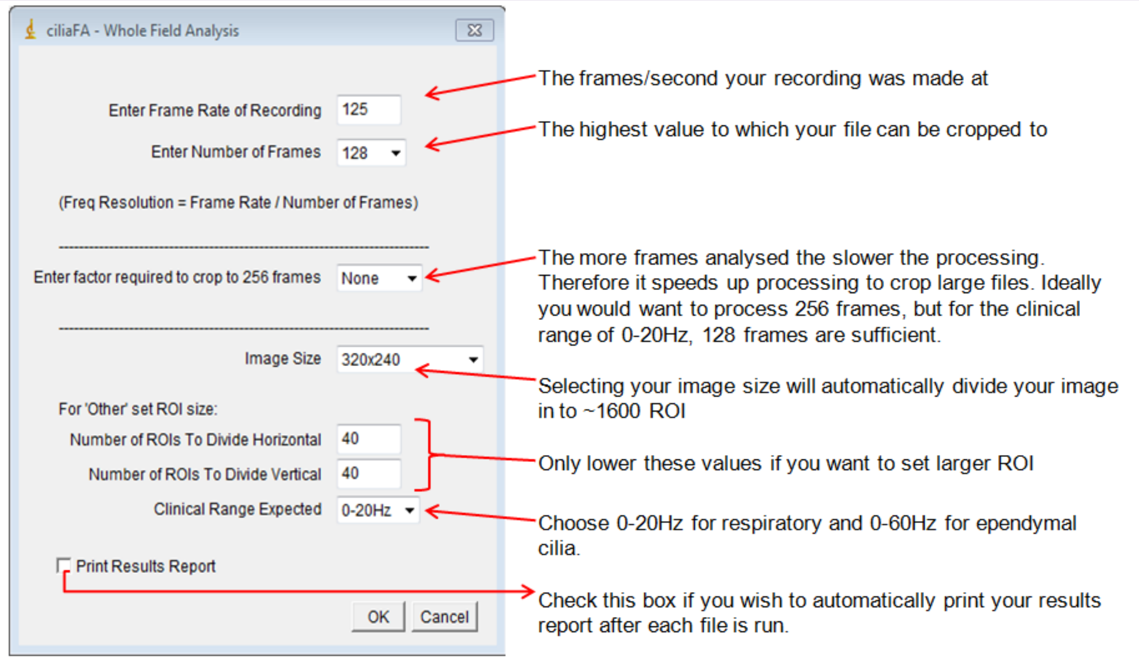

3.4 Browse and select the folder that contains the AVI files you want to be analysed:

3.5 Analysis takes approximately 3 minutes per AVI file depending on your processor speed. Do not use your computer while ciliaFA is running as this can interfere with processing. It performs best if left to run overnight.

Wait until this message appears:

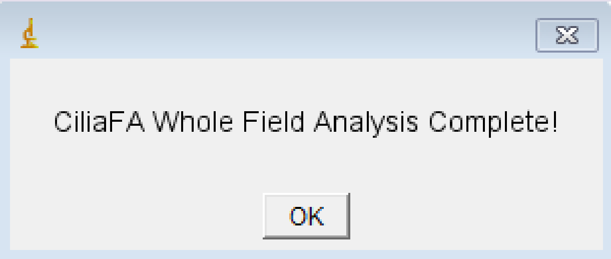

TIPS ON IMAGE AQUISTION THAT CAN IMPROVE YOUR ANALYSES:

Limitations of the software depend largely on image quality and so here are some easy steps that can improve the quality of your image and your results:

1) Use the smallest pixel size available that shows sharp, clear cilia movement.

2) Use a high power objective or crop your image so that the motile cilia fill the image. The more 'dead space' in your image the less ROI are being used for the analyses.

3) For high frequency analysis, we recommend that the microscope bulb is connected to a stable power source of at least 110 volt AC/ 60 Hz, lower voltages will encourage the bulb to flicker within the CBF range and this will enhance background noise. We also recommend that the image is not subject to down-stream processing, such as enhanced pixel gain, as this will also enhance background noise.

4) Capture at least double the number of frames to the frame rate of recording. We have used the ciliaFA software to examine .avi files of different lengths, captured at different frame rates. We found that videos captured using high speed video cameras that capture at rates at least 120 frames per second with a length of 128 frames (to give a frequency resolution of 0.94) will give valid data. These settings capture an appropriate number of ciliary beat cycles to accurately average the CBF; the lower the frequency resolution (i.e. the more frames captured at this frame rate), the greater the accuracy of CBF.

5) To allow the videos to be used for beat pattern analysis high speed video cameras that capture at rates exceeding 250 frames per second should be used. The ciliaFA software has the capability to crop frames from large files to speed up the calculation of frequency without reducing the frequency resolution.

4. Interpreting your results

Your results will be saved as:

1) an Excel file
2) a PDF of only the "Results Report". This will be automatically printed if you checked the 'Print' box.

The Excel file allows you to examine the data in many ways:

SHEET 1. THE "RESULTS REPORT":

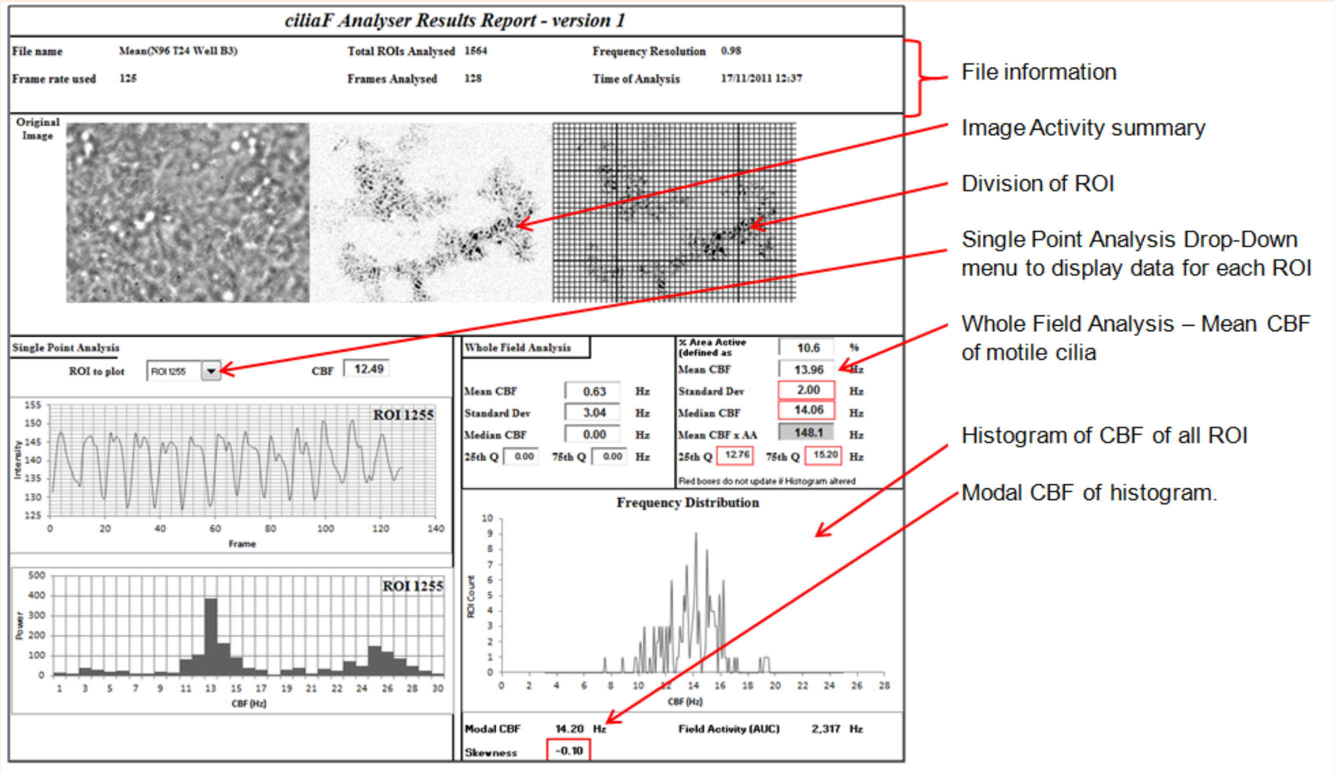

If you would like to examine the raw data – and maybe delete a particular frequency that you think maybe background interference then 'unhide' the "FFT Results" sheet. Delete the frequency data from the histogram. The overall data on the results report will be updated (except the data that appear in red boxes).

SHEET 2. THE COLOUR CHART:

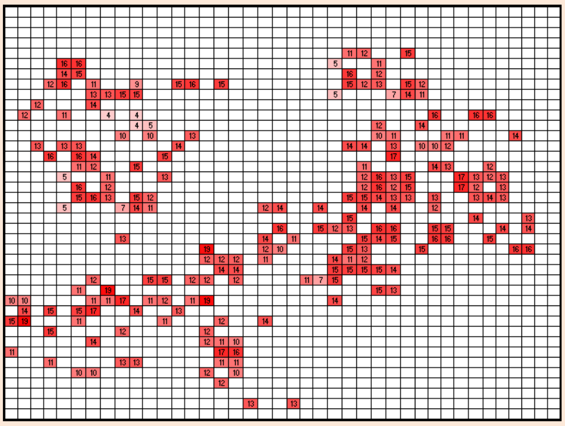

This shows how the CBF of each ROI as the image was divided up. To find out what number corresponds to each ROI:

1) Open your .avi in ImageJ and
2) Go to "**ROI Manager**".
3) Select "**Open**"
4) Open the "RoiSet.zip" file included in the ciliaFA Results folder that contains your results.
5) Check "**Show All**" and "**Edit Mode**".

5. Troubleshooting

Common errors:

**Error**	**Solution**
Incorrect File name saved	All .avi file names must start with a letter not a number, must be no longer than 16 characters and contain no punctuation.
Macro error after Excel opens:<br>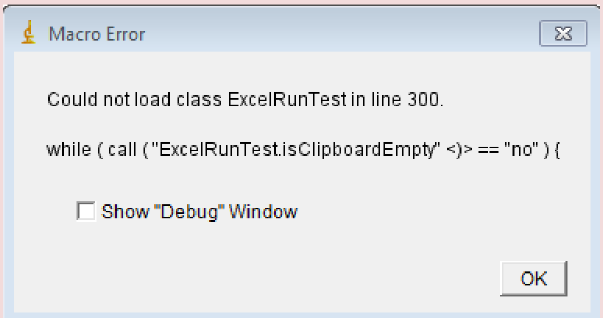 	**1.**The Java file is not installed properly<br><br>Check the **“ExcelRunTest.class”** file appears in your ciliaFA folder. If not go to section 2.6-2.7 of these setup instructions<br><br>If the “**ExcelRunTest.java”** file does not have the .java file extension showing, check the following:<br><br>Go to Folder Options and make sure the option to ‘show file extensions’ is checked.<br><br>**2.** If the software has previously been setup and run, but is now not functioning properly try restarting your computer.
Error message after data is pasted into Excel:<br>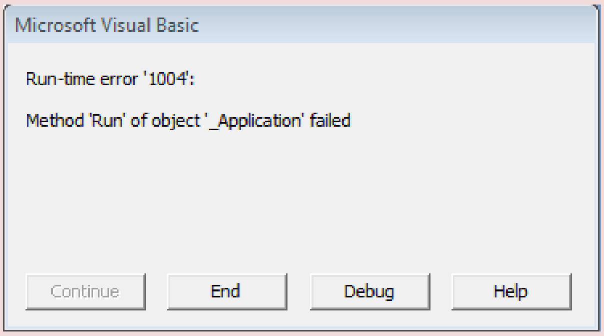 	The Analysis Toolpak and Analysis Toolpak VBA Addin have not been correctly installed in Excel. Go to section 1.2 of these setup instructions.
